# Patients’ Experiences Using a Mobile Health App for Self-Care of Heart Failure in a Real-World Setting: Qualitative Analysis

**DOI:** 10.2196/39525

**Published:** 2023-08-15

**Authors:** Ifeanyi Madujibeya, Terry A Lennie, Jamie Pelzel, Debra K Moser

**Affiliations:** 1 Research and Interventions for Cardiovascular Health Heart Program College of Nursing University of Kentucky Lexington, KY United States; 2 Center for Nutritional Sciences College of Nursing University of Kentucky Lexington, KY United States; 3 Heart and Vascular Center CentraCare St Cloud, MN United States

**Keywords:** heart failure, patients’ experiences, experience, satisfaction, facilitator, mobile health apps, mobile app, health app, app feature, mobile health, cardiology, cardiovascular, patient care, self-management, patient, heart, mHealth, self-care, medication, performance, feedback, personalized

## Abstract

**Background:**

Publicly available patient-focused mobile health (mHealth) apps are being increasingly integrated into routine heart failure (HF)–related self-care. However, there is a dearth of research on patients’ experiences using mHealth apps for self-care in real-world settings.

**Objective:**

The purpose of this study was to explore patients’ experiences using a commercially available mHealth app, OnTrack to Health, for HF self-care in a real-world setting.

**Methods:**

Patient satisfaction, measured with a 5-point Likert scale, and an open-ended survey were used to gather data from 23 patients with HF who were provided the OnTrack to Health app as a part of routine HF management. A content analysis of patients’ responses was conducted with the qualitative software Atlas.ti (version 8; ATLAS.ti Scientific Software Development GmbH).

**Results:**

Patients (median age 64, IQR 57-71 years; 17/23, 74% male) used OnTrack to Health for a median 164 (IQR 51-640) days before the survey. All patients reported excellent experiences related to app use and would recommend the app to other patients with HF. Five themes emerged from the responses to the open-ended questions: (1) features that enhanced self-care of HF (medication tracker, graphic performance feedback and automated alerts, secured messaging features, and HF self-care education); (2) perceived benefits (provided assurance of safety, improved HF self-care, and decreased hospitalization rates); (3) challenges with using apps for self-care (giving up previous self-care strategies); (4) facilitators (perceived ease of use and availability of technical support); and (5) suggested improvements (streamlining data entry, integration of apps with an electronic medical record, and personalization of app features).

**Conclusions:**

Patients were satisfied with using OnTrack to Health for self-care. They perceived the features of the app as valuable tools for improving self-care ability and decreasing hospitalization rates. The development of apps in collaboration with end users is essential to ensure high-quality patient experiences related to app use for self-care.

## Introduction

The cost of heart failure (HF) in the United States is projected to double by 2030, exceeding US $69 billion per year [1]. More than 68% of this cost is attributed to frequent rehospitalizations for symptom exacerbations and comorbidity management [2]. In addition, nearly 25% of patients with HF are rehospitalized within 30 days of discharge [3,4].

Patients who engage in self-care behaviors are 50% less likely to be rehospitalized within 30 days of discharge [5]. Self-care is a naturalistic decision-making process that promotes actions to maintain physiological balance and well-being (self-care maintenance), enhances recognition of symptoms (symptom perception), and impacts patients’ responses to symptoms (self-care management) [6]. Self-care behaviors reduce the risks of physiological instability and increased symptom burden [7]. However, patients with HF find it difficult to engage in self-care behaviors that require complex skills, such as symptom monitoring and interpretation [8].

The ubiquitous and cost-effective nature of mobile devices has prompted the development of consumer-facing mobile health (mHealth) technologies such as mHealth apps. mHealth apps may improve patients’ abilities to engage in self-care by providing automated self-care feedback and facilitating patient-provider collaboration in HF self-care. Recent reviews [9-11] identified more than 34 commercially available mHealth apps for improving HF self-care. Little is known about patients’ experiences using these apps for self-care of HF in real-world settings. Previous researchers [12-16] had examined patients’ experiences of using mHealth apps for self-care and concluded that patients found apps helpful and were satisfied with them for self-care. However, all of the mHealth apps used in the interventions were research-grade apps that were not available for consumers, which reinforces the call by the American Heart Association to examine the efficacy of commercially available mHealth apps in improving HF outcomes [17].

In real-world settings, patients are expected to engage with mHealth apps for a sufficient period to achieve targeted self-care behaviors and health outcomes [18]. However, patient engagement with mHealth apps in real-world settings is difficult to maintain and tends to decrease over time [19]. The insight gained from the experiences of patients who used mHealth apps for a long period may identify factors associated with long-term patient engagement with mHealth apps. However, previous studies [12-16] that described patients’ experiences using mHealth apps were characterized by a short duration of interventions, ranging from 1 to 4 months. The studies’ durations may have been insufficient to provide insight into long-term patient engagement with apps in real-world settings.

Given the paucity of evidence, the European Society of Cardiology recommended that future studies should focus on long-term patient engagement with mHealth apps in real-world settings instead of conducting more randomized controlled trials to demonstrate the feasibility of mHealth interventions in patients with HF [20]. A study of patients’ experiences using mHealth apps in a real-world setting is warranted. Thus, the purpose of this study was to explore patients’ experiences using a commercially available mHealth app, OnTrack to Health, for HF self-care in a real-world setting.

## Methods

### OnTrack to Health Program

**Figure 1:**
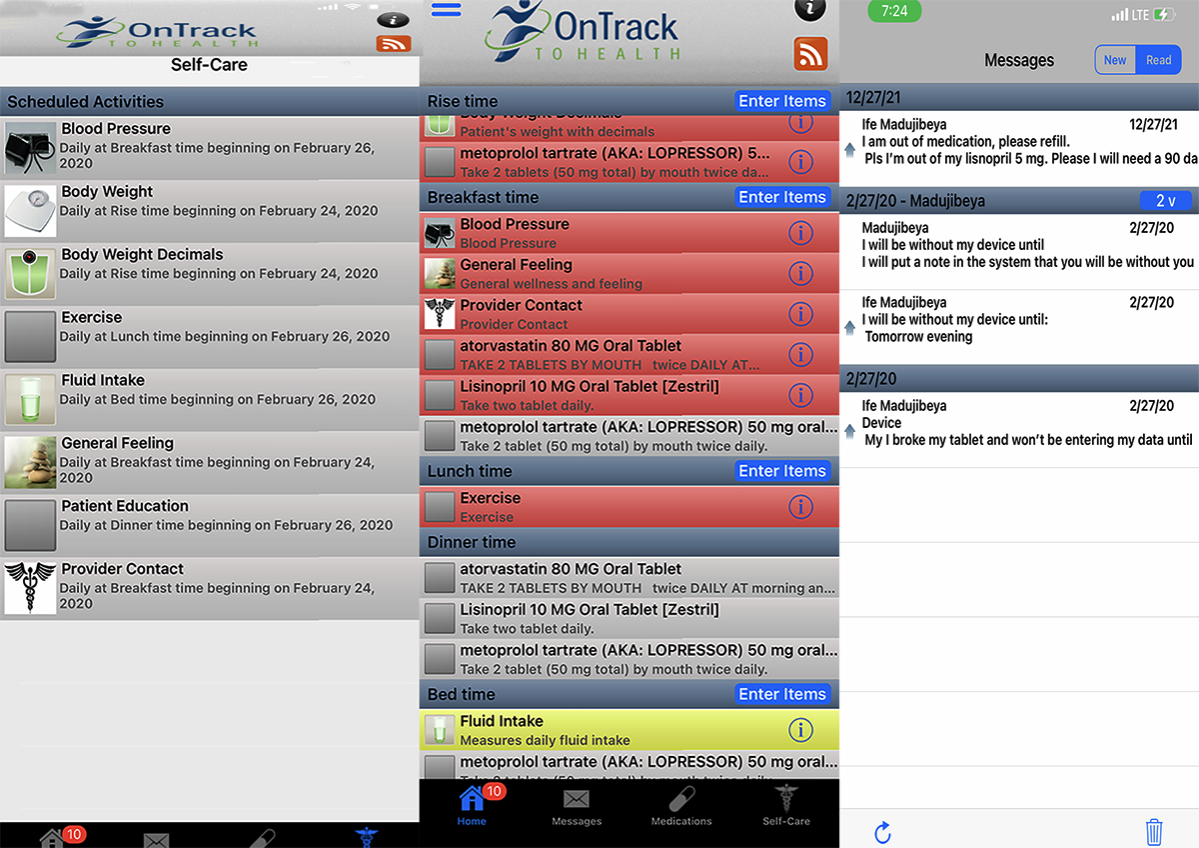
OnTrack to Health app.

OnTrack to Health app [21] was developed in collaboration with patients with HF, their caregivers, and health care providers. In 2012, OnTrack to Health program was deployed as part of the standard of care in a HF outpatient clinic in Minnesota. OnTrack to Health (Figure 1) is a subscription-based commercial app for HF self-care that includes interfaces for invasive and noninvasive remote monitoring. The app is comprised of a clinician web portal to access and monitor transmitted and real-time patients’ health parameters stored on the cloud; a mobile app that includes interfaces on 42 self-care measures; and portable consumer-facing digital devices. The mobile app has the following six main components: (1) a home screen that is personalized to display the 42 self-care features such as daily medications, exercise, sodium intake, general health status, and telemonitored physiological variables that were assigned to patients based on their tailored needs; (2) invasive remote monitoring interfaces for CardioMEMS hemodynamic sensor and left ventricular-assisted devices; (3) a medication tab that shows an active medication list and direction for taking each medication; (4) a secure messaging screen for communication between patients and their providers; (5) self-care activities screen that displays each assigned self-care instruction and provides ongoing feedback to the patient on their adherence trends; and (6) a patient education tab populated with general and personalized HF information.

OnTrack to Health was prescribed to patients with HF at high risk for hospitalization because of poor self-care. Self-care was assessed based on patients’ self-report during routine visits. Patients were considered at risk for hospitalization if they reported nonadherence to recommended self-care activities [5,7,22]. Patients who consented to use the app were trained on each feature of the app. The consent includes a plan to use patients’ deidentified data in future studies. A cardiologist decided which self-care behaviors were recommended (from medications, body weight, blood pressure, exercise, and fluid intake) and the frequency of monitoring (eg, daily for blood pressure and body weight) according to the patient’s clinical characteristics and self-care needs. Patients were instructed to log into the app to check the completion of their assigned self-care activities using their mobile devices. Patients received an alert on their mobile devices when assigned self-care activities were due. The completed assigned tasks were transmitted via a cellular network to a secured database server. Patient patterns of adherence to the tasks could be assessed in tabular and graphic formats via the clinician web portal. Those who did not complete 1 or more of the assigned self-care activities after 24 hours received a follow-up call or secure text message from nursing staff dedicated to monitoring the clinician web portal.

If any of a patient’s monitored parameters were outside of a predefined range, a system alert would be triggered and a trained HF nurse monitored the clinician web portal for these alerts during regular business hours. Notifications received during the weekend were read on Monday. The nursing staff followed up with patients via phone calls or secure messages to validate parameters that deviated from the expected range. All clinically relevant alerts were directed to a cardiologist. When participants were unable to transmit their monitored measures, such as when hospitalized, traveling to locations with a poor cellular network, or experiencing unresolved technical issues, they were required to notify the nursing team, and monitoring would be suspended for the period.

The subscription cost, including iPads and data plans, was covered by the clinic for the first 60 days. After 60 days, patients covered the subscription cost and could download the app to their own mobile devices that had a data plan. Patients were unenrolled from the program when they stopped seeing clinicians from the clinic or improved their self-care behavior to the extent that the app was no longer necessary. At this point, self-care was measured based on self-report and self-care adherence level captured by the app.

### Design

This study was a qualitative analysis of data collected by open-ended questions guided by a survey questionnaire (Multimedia Appendix 1) administered via the secure messaging feature of the OnTrack to Health app to patients who were using the app in 2016. The survey also included patient satisfaction, measured with a 5-point Likert scale (1=poor to 5=best). Trained clinic staff checked all the responses for accuracy and completeness, and followed up with patients who gave incomplete responses or patients who requested more information to ensure understanding of the survey’s questions. All the data were stored in OnTrack to Health databases.

### Data Analysis

The data were extracted from the databases and uploaded to a qualitative data analysis software, Atlas.ti (version 8) for analysis. The 3 phases (preparation, organizing, and reporting) of deductive content analysis outlined by Elo and Kyngäs [23] were used to analyze the qualitative data. In the preparation phase, the data were read multiple times to gain a depth understanding of the content and to identify possible coding units. In the organizing phase, line-by-line coding was carried out by grouping the data into clusters of information and assigning labels to the clusters (codes). The list of codes was combined into potential themes to describe the patients’ survey responses to using OnTrack to Health for HF self-care. The potential themes were refined to ensure that data within each theme were distinctive. In the reporting phase, the data were summarized using illustrative quotes along with the frequency of patients who reported specific codes.

Patients were identified in the quotes as long-term users (used OnTrack ≥1 year) or short-term users (used OnTrack to Health <1 year). We used a 1-year cutpoint in line with the recommendation for studies of patients who used mHealth in real-world settings for 1 or more years [20]. Two individuals independently conducted the initial analysis. Any disagreements during the analytic process were discussed until a consensus was reached. The codes were reviewed by all authors. An external auditor blinded to the research goals and questions reviewed all the codes and their interpretations [24,25].

## Results

### Overview

The patient demographics, clinical characteristics, and mHealth use data were summarized using the appropriate descriptive statistics (mean, median, and frequency) for the level of measurement. The study was guided by the Consolidated Criteria for Reporting Qualitative Research (COREQ). COREQ is a 32-item checklist to guide reporting of qualitative studies [26].

Out of 87 patients who received the survey, 75 (86%) read it and 23 (26%) responded. Table 1 summarizes the demographic and clinical characteristics of the respondents. The median patient age was 64 (IQR 57-71) years. The majority (17/23, 74%) of patients were men. Patients had used OnTrack to Health for a median 164 (IQR 51.2-640) days before the survey.

**Table 1:** Patient demographic and mHealth usage characteristics (n=23).

Variables		Values
Age (years), median (IQR)		64.0 (57.0-70.50)
Sex (male), n (%)		17 (74)
**Symptom monitoring modalities**		
	Noninvasive (daily weight and blood pressure), n (%)	19 (75)
	Noninvasive and CardioMEMS, n (%)	3 (25)
	Noninvasive and left ventricular assisted device, n (%)	1 (4.3)
	Duration of app’s use before the survey (days), median (IQR)	164.0 (51.2-639.9)
**User category, n (%)**		
	Number of long-term users	8 (35)
	Number of short-term users	15 (65)
	Number of medications taken daily, median (IQR)	9.0 (7.0-10.6)

### Ethics Approval

This study was approved by the institutional review board of the University of Kentucky (62205). This investigation conformed to the principles outlined in the Declaration of Helsinki.

### Patient Satisfaction With Using OnTrack to Health

All patients rated their experience related to the app use as excellent. They stated that they would recommend the app to other patients with HF. One patient stated,

I would wholeheartedly (no pun intended) rate OnTrack a 5 out of 5. The benefits of using OnTrack are beyond measure as far as my wife and I are concerned. I would absolutely recommend this program to other patients with heart failure.

Long-term user, 19.6 months

### Themes

#### Overview

The following five themes were identified from the data: (1) features that enhanced self-care of HF ability, which included medication tracker, graphic performance feedback and automated alerts, and secured messaging features; (2) perceived benefits of using the app, which included assurance of safety, improved self-care, and decreased hospitalization; (3) challenges to adopting the app for self-care, which included giving up previous self-care strategies; (4) facilitators that included perceived ease of use, availability of technical support; and (5) suggested improvements, which included, streamlining data entry, integration of the app with their electronic medical record and personalization of app features.

#### Theme 1: Features That Improved HF Self-Care Ability

##### Medication Tracker

The majority of patients (14/23, 61%) stated that the medication tracking features of OnTrack to Health (medication reminder alarm and visual display of medication adherence) and availability of their current medications list and instruction for taking them on the app’s medication tracker helped them take their medication as prescribed. One patient stated,

It seems to be a good tool for us to keep track of the medicines and then you can see what we are doing with them too.

Long-term user, 22.3 months

Another patient stated,

The program (OnTrack to Health) reminds me to take my medications on time to have a better chance of staying out of the hospital.

Short-term user, 2.3 months

##### Graphic Performance Feedback and Automated Alerts

Patients perceived OnTrack to Health as a helpful tool for facilitating their ability to monitor their symptoms. Among the patients, 44% (10/23) indicated that the app’s ability to display skipped self-care activities (graphic performance feedback) and the app’s automated reminder alerts motivated them to improve their self-monitoring ability and to take appropriate actions to address changes in HF status. For instance, 1 patient stated,

It (OnTrack to Health) makes me conscious of checking my weight. I do occasionally forget to check my weight when getting up in the morning, but when I see it on OnTrack I tend to remember the next day. It reminds me daily how important it is to check my weight, otherwise, I do not think I would be doing it today.

Long-term user, 33.2 months

Another patient said,

I used to just keep papers on the counter with my weight and I like this (OnTrack) better and if I see my weight go up, I send a note to a nurse and there is help.

Short-term user, 3.07 months

##### Secured Messaging Feature

Moreover, 78% (18/23) of the patients stated that the app’s secured messaging feature was very efficient in facilitating communication and connection with their health care teams. Patients indicated that the secure messaging system helped them get quick responses from their health care providers, without spending time on telephone calls or driving long distances to the clinic to address their concerns. For example, 1 participant stated*,*

If I have symptoms and need a health care provider to contact me, I have the assurance that with one touch of my finger, someone will soon be in touch. We would feel somewhat abandoned without OnTrack.

Long-term user, 19.6 months

Another patient (long-term user, 34.8 months) stated, “I receive quick responses, no frustrating phone calls.”

##### HF Self-Care Education

Out of the 23 patients, 2 (9%) perceived the self-care educational feature of OnTrack to Health as a reliable source of information about HF. They expressed the view that accessing the information had made them more informed about better managing their HF. One participant stated,

OnTrack answers my questions and makes me feel safe. It has good information on the site which has helped me to cut the stress in my life. I just ate with family recently and the restaurant fixed my food with no salt.

Short-term user, 3.1 months

#### Theme 2: Perceived Benefits of Using OnTrack to Health

##### Assurance of Safety

The majority of patients (12/23, 52%) stated that the sense of connectedness with their health care providers and remote monitoring of their symptoms provided them assurance of safety and peace of mind. One patient stated,

For my wife, the iPad provides peace of mind that someone other than herself is monitoring my health daily.

Long-term user, 19.6 months

Another patient stated,

We feel that we are connected to the clinic and if we have any concerns, they are at the other end will help. It gives us lots of peace of mind. We feel safe even four hours away. We feel we can send a message and get advice or support.

Short-term user, 6.8 months

##### Improvement in HF Self-Care

Patients perceived HF self-care, such as taking their medication as prescribed, adhering to restricted salt diets, and monitoring and managing their symptoms as complex and challenging. All patients stated that their enrollment in the OnTrack to Health program and using the app daily had simplified 1 or more aspects of their self-care activities, making them easier to perform as instructed by their providers. For example, 1 patient stated,

The benefits are not trying to remember from day to day when to take medications. It can be confusing without the use of this program (OnTrack to Health App).

Long-term user, 22.3 months

##### Decreased HF Rehospitalization

Among the patients, 39% (9/23) stated that OnTrack to Health was instrumental in helping them improve their health and decrease their hospitalization rates. For example, 1 patient stated,

I believe OnTrack is instrumental in keeping me out of the hospital as much as possible, the benefits of using OnTrack are beyond measure.

Short-term user, 6.3 months

Another patient stated,

The only time I’ve been in the hospital since I been OnTrack was for a bloody nose.

Long-term user, 22.3 months

#### Theme 3: Challenges to Using an App for Self-Care

##### Giving Up Previous Self-Care Strategies

Out of the 23 patients, 2 (9%) identified the challenge of changing their previous self-care routine as a barrier to the effective use of OnTrack to Health for self-care activities. However, patients were confident that their ability to use the app more effectively would improve over time. One of the patients stated,

I am so used to doing things with my pills in a certain ritual and to change is hard, but in time it should be better.

Short-term user, 2.1 months

##### Forgetfulness With Using App

Out of the 23 patients, 2 (9%) reported initial challenges with remembering to use the app as instructed; however, they were determined to incorporate the app into their daily self-care routine. One of the patients stated,

It is just for us, old people, to remember to use the iPad, as we are not used to doing things this way. I know I forget and have to enter it when I think of it.

Short-term user, 2.1 months

#### Theme 4: Facilitating Conditions

##### Ease of Use

Among the patients, 13% (3/23) stated that OnTrack to Health was user-friendly and easy to use. One patient stated,

The app is very convenient and easy to use, with one click of a button, you can get help when you need it.

Short-term user, 1.9 months

##### Ready Availability of Technical and Medical Support

Some of the patients (4/23, 17%) stated that they were satisfied with using OnTrack to Health for HF self-care because of the prompt technical and medical support they received from OnTrack support and their health care providers, respectively. As 1 patient indicated,

Both OnTrack support and the clinic have always returned any messages I have left asap. They always have the answers that I need. Working and teaching at an elementary school make it very hard to be on the phone all day to speak to someone. As well as the fact I live 70 miles away from the clinic.

Long-term user, 32.1 months

#### Theme 5: Suggested Improvements

##### Streamlining Data Entry Process

Out of the 23 patients, 3 (13%) stated that they found it challenging to remember to enter their completed self-care activities on OnTrack. Patients suggested approaches to streamline the data entry processes, such as automating the data entry process and granting users the ability to enter omitted entries whenever users remembered the omissions. One patient suggested,

Sometimes we messed up on entries, I took the correct medication, just sometimes I goofed on entering them. The only thing I would like to be able to do is to be able to go back to enter taking a med when I have forgotten.

Short-term user, 1.6 months

##### Personalization of App Features

Among the patients, 8.9% (2/23) suggested that patients should be granted the ability to personalize some of the app’s functionalities to fit users’ personal needs. Two features that patients stated they would like to customize were reminders and medication schedules. For instance, 1 patient stated,

I mentioned alarms; I would like them to be tied to the groups (assigned self-care activities). I understand that meds have to be taken at specific times of my day; It will be good for a user to adjust the time a group has to be taken.

Long-term user, 33.2 months

Another patient stated,

Change the times you go to the next day’s medication. I don’t always go to bed before you change to the next day, so I don’t take some of my night medicines until 2 or 3 in the morning.

Short-term user, 6.3 months

##### Integration of Apps With Electronic Medical Records

One patient (long-term user, 19.6 months) recommended integrating OnTrack to Health to the patient portal to give patients access to their medical health records via the app. The patient stated, “The only suggestion would be to somehow link it (OnTrack to Health) to MyChart.”

## Discussion

### Principal Findings

We conducted a qualitative analysis to describe patients’ experiences using an HF self-care app (OnTrack to Health) to support self-care in a real-world setting. All patients were highly satisfied with using the app for self-care and would recommend it to other patients with HF. Patients perceived the features of OnTrack to Health as valuable tools to communicate with health care providers, improve self-care, and decrease HF hospitalization rates. In addition, patients made suggestions about what could be improved in the app. They recommended integrating the app into an electronic medical record and giving users the ability to customize certain features of the app.

### Comparison With Previous Work

The high level of patients’ satisfaction with using OnTrack to Health for self-care is consistent with findings in previous studies [14,16,27,28] that described patients’ experience using mHealth apps for self-care. One of the studies involved a 6-month intervention in which an mHealth app, CardioCoach, was to facilitate HF symptom monitoring and titration of HF medications based on the reported symptoms. At the end of the intervention, all the patients reported high levels of satisfaction with using the app for their medication management. Thus, the findings from these studies suggest that patients with HF are satisfied with using mHealth apps for self-care. Our study extended the evidence to indicate that the high level of satisfaction with mHealth apps was not limited to patients who used the apps in research settings but may include both short-term and long-term users in real-world settings.

The positive patient experiences with the OnTrack to Health app may be related to the perceived usefulness of the app’s features in improving patients’ self-care ability. Patients associated the medication tracking, graphic performance feedback, automated alerts, secured messaging, and HF self-care education features of the app, with simplifying the complexity of self-care activities, making them easier to perform. Consequently, a decrease in hospitalization rates was attributed to improvement in self-care as a result of the app use. These findings reflect the ability of persuasive functionality of mHealth apps to target behavior change through the provision of effective self-care support, such as automated reminders and self-care adherence feedback. Such support may improve patients’ self-efficacy to engage in self-care by decreasing the experience and skills required to perform complex self-care activities [29].

The perceived usefulness of OnTrack to Health features in improving self-care and decreasing rehospitalization rates may account for long-term patient engagement with the app. Recent investigators showed that patients usually discontinue the use of an mHealth app within 30 days of installing the app [30,31]. The lack of perceived usefulness of app features to achieve targeted behavior change was the main reason associated with discontinuing use [11,30,31]. Considering that all the patients in our study who used OnTrack to Health for a duration of 12 to 36.3 months (long-term users) perceived the app as a useful tool for improving self-care, the perceived usefulness of the app might be associated with sustained engagement in the long-term user.

The secured messaging was an essential app feature of OnTrack to Health identified by most of the patients as useful for improving self-care. Most of the patients indicated that the secured messaging feature of the app was very efficient in facilitating their communication and connection with their health care team. Patients perceived the feature as an efficient method to communicate with their health care providers. These findings reflect the experience of veterans who used a secure messaging tool embedded in a web-based patient portal to communicate with their providers [32]. The veterans highlighted how secured messaging provided them with an effective platform to communicate and collaborate with their health care providers in managing their health conditions. Similar to our findings, the veterans stated that secure messaging eliminated the need for frustrating phone calls or driving long distances for face-to-face interaction [32]. Thus, mHealth apps with secure messaging that are fully integrated with the patient’s health care providers may be important for long-term usage and vital for promoting patient-provider collaborations in improving patients’ self-care.

OnTrack to Health’s developers used a user-centered design principle by including feedback from patients, nurses, and cardiologists in every stage of the app’s development. The collaboration between end users and developers at each phase of the app’s development ensured not only that features that were important to end users were included in the app but were designed in such a way that fulfilled the expectations of patients, their caregivers, and health providers, leading to the best patients experience.

In addition, patients’ positive experiences related to OnTrack to Health use may be explained by the app’s ease of use. OnTrack to Health has a user-friendly interface. Patients could access all features of the app from the home screen, which minimizes the need to navigate through multiple screens to complete a self-care task. None of the patients in the survey suggested any difficulty using the app. Some patients attributed their satisfaction with the intervention to the app’s ease of use. Similarly, investigators in previous mHealth interventions in which patients had positive experiences reported that perceived ease of use was a major factor in patient positivity [13,15,27,28,33-36]. Even older adults who considered themselves technologically inept have reported that they found it easy to use mHealth apps for self-care [13]. A review of 106 mHealth apps identified ease of use as a major determinant of patient satisfaction using mHealth apps [37]. Our findings add to this literature, suggesting that ease of use is a critical characteristic of mHealth apps that will influence the patient experience of using mHealth apps for self-care in a real-world setting.

Previous investigators reported that patients experienced various degrees of technical difficulty when using mHealth apps for self-care [12-14,16,19,33,38]. In one of the studies, 22% (12/54) of the participants in the intervention group did not start an mHealth intervention as a result of the participants’ inability to navigate the mHealth app used in the intervention [38]. Investigators also reported patients’ frustration with mHealth interventions as a result of experiencing technical difficulties with apps [12]. In contrast, none of the patients who used OnTrack reported experiencing technical problems with the app. It is unlikely that OnTrack to Health program was completely free of technical issues. The patients probably experienced technical issues but considered the issues too insignificant to warrant reporting in the survey [27]. Furthermore, after enrollment in OnTrack to Health program, patients were trained on all features of the app. Technical support was readily available to help patients resolve technical issues. Ease of use, tailored patient training on the app’s features, and availability of technical support were associated with decreased incidence of technical issues in previous mHealth interventions [14,27,39] and may likely explain the lack of reports of technical problems in our study.

In addition, previous investigators suggested that the cost of mHealth interventions could be a barrier that may hamper patient engagement with the interventions [40,41]. In contrast, none of the participants in our study identified the cost of intervention as a barrier. Dang et al [14] found that patients with HF were willing to pay a small monthly fee to continue using the mHealth interventions they perceived useful for improving HF outcomes. Considering that the long-term users in our study reported that OnTrack to Health was useful for improving HF outcomes, the perceived usefulness of OnTrack to Health may be associated with the lack of sentiments on cost.

Personalization of app features or tailoring functionality to specific patients’ needs is an important factor associated with long-term patient engagement with mHealth apps [37,42]. Similar to our findings, the investigators in a previous review [37] identified automated alerts and reminders as features of mHealth apps that patients would like to customize. Patients were more likely to use mHealth apps that generate tailored alerts compared to apps that did not [43,44]. Automated alerts and reminders have been integrated into mHealth interventions in patients with HF [15,27,36,39,40,45-48]. However, none of the studies described patients’ perceptions of the reminders. In contrast, our findings suggest that patients may perceive the ability to customize automated reminders as essential for using mHealth apps for self-care.

### Strengths and Limitations

All the patients in our study were current users of OnTrack to Health during the survey, which minimizes the likelihood of a recall bias in our findings. Recall bias occurs when a participant could not accurately recall a previous experience. For example, the investigative team in an mHealth intervention, Health Buddy [15], interviewed patients about 6 months to 2 years after the intervention was completed. The team suggests that some information was lost as a result of the participants’ inability to accurately recall some aspects of their experience of using Health Buddy. Thus, the lack of lapse in time between OnTrack to Health’s use and our survey could add to the validity of our findings.

Our study has some limitations. First, our survey has a low response rate of 26% (23/87). Although there was a lot of similarity in the responses, a higher response rate may have offered additional insight into the experience. Second, the administration of our survey via OnTrack to Health’s secured messaging could lead to a nonresponse bias in our findings. A nonresponse bias occurs when survey data are limited to respondents who differed from the nonrespondents in sample characteristics that could affect findings [49]. Patients who used OnTrack to Health less frequently or had stopped using the app before the survey might be less likely to respond to the survey. Their experiences of using OnTrack to Health might differ from those of the respondents, resulting in nonresponse bias. However, the result of our nonresponse bias analysis (Multimedia Appendix 2) indicates that the duration and frequency of the app’s use were not associated with the patients’ odds of responding to the survey. Third, although member-checking or study participants’ validation of findings is considered a critical technique for establishing the credibility of findings [25,50], we did not conduct member-checking as our data were collected in 2016.

### Conclusions

Patients were satisfied with using OnTrack to Health for self-care in a real-world setting. They perceived the app as a valuable tool for improving their self-care ability and decreasing their hospitalization rates. Development of mHealth apps in collaboration with end users, proper training of patients on app features, tailoring of app features to specific patient needs, and mHealth apps with secure messaging that are fully integrated with the patient’s health care providers are essential to ensure the best patient experience related to the use of mHealth apps for self-care.

## Appendix

Multimedia Appendix 1 Questionnaire guide.

Multimedia Appendix 2 Nonresponse bias analysis.

## Abbreviations

COREQ: Consolidated Criteria for Reporting Qualitative Research
HF: heart failure
mHealth: mobile health
