# Supplementary material for: Patients’ Experiences Using a Mobile Health App for Self-Care of Heart Failure in a Real-World Setting: Qualitative Analysis
Source: JMIR Form Res. 2023 Aug 15;7:e39525. doi: 10.2196/39525 (PMC10466157; doi:10.2196/39525)
Supplement: Multimedia Appendix 1 [file formative_v7i1e39525_app1.docx]

Thank you for being a part of the OnTrack to Health (iPad) program and for taking ownership in managing your health given a difficult diagnosis. CentraCare plans to continue to use OnTrack with more patients and would like your feedback as to what you most like about using OnTrack, what you would change, and what you would like to see added that would best help you manage your health. Your feedback on the following questions would be greatly valued!

1- How would you rate your experience of using OnTrack to stay connected with your clinical team on a scale of 1-5 where 5 is the best?

2- Do you believe OnTrack is helping to keep you out of the hospital as much as possible?

3- Would you recommend other patients with heart failure use OnTrack?

4- How would you describe the benefits of using OnTrack and what you get out of using the program?

5- Do you have any ideas you would like to share about how OnTrack could be enhanced to provide even more value or changes we could make to help keep you on track?
