# Supplementary material for: Patients’ Experiences Using a Mobile Health App for Self-Care of Heart Failure in a Real-World Setting: Qualitative Analysis
Source: JMIR Form Res. 2023 Aug 15;7:e39525. doi: 10.2196/39525 (PMC10466157; doi:10.2196/39525)
Supplement: Multimedia Appendix 2 [file formative_v7i1e39525_app2.docx]

Binomial logistic regression analysis was conducted to determine what demographic (age, sex) and app use ( duration of app use before survey in months, number of times a patient interacted with the app per month) variables best predict whether a patient would respond to the survey.

Model equation:

log(P/1-p ) = βintercept +β(Duration_BeforeSurveyMonths)+ β(num_usagePerMonth ) + β(Age) + β(Sex)

P : Probability of responding to the open-ended surveys

β: Coefficients

Duration_BeforeSurveyMonths : Duration of time a patient (in months) used the app before the survey.

num_usagePerMonth: Average number of times a patient interacted with the app per month

Age : patient’s age in years.

Sex: Indicates whether a patient is a male or female.

Table S1. Predictors of patients’ response to the open-ended surveys

|  | **response Coded** | | |
| --- | --- | --- | --- |
| *Predictors* | *Odds Ratios* | *CI* | *p* |
| (Intercept) | 0.01 | 0.00 – 0.16 | **0.006** |
| Duration_BeforeSurveyMonths^a^ | 1.04 | 0.99 – 1.09 | 0.111 |
| num_usagePerMonth^b^ | 1.00 | 1.00 – 1.00 | 0.983 |
| Age | 1.07 | 1.02 – 1.13 | **0.018** |
| Sex [female] | 0.35 | 0.10 – 1.02 | 0.067 |
| Number of patients | 87 | | |

^a^ Duration_BeforeSurveyMonths : Duration of time a patient (in months) used the app before the survey.

^b^ num_usagePerMonth : Average number of times a patient interacted with the app per month
